# Effect of rearing style on the development of social behaviour in young ravens (*Corvus corax*)

**DOI:** 10.1111/eth.13010

**Published:** 2020-03-18

**Authors:** Palmyre H. Boucherie, Christian Blum, Thomas Bugnyar

**Affiliations:** ^1^ Department of Cognitive Biology University of Vienna Vienna Austria; ^2^ Haidlhof Research Station University of Vienna and University of Veterinary Medicine Vienna Vienna Austria

**Keywords:** corvids, hand‐raising, ontogeny, social competence, social experience, social relationships

## Abstract

Early social experiences can affect the development and expression of individual social behaviour throughout life. In particular, early‐life social deprivations, notably of parental care, can later have deleterious consequences. We can, therefore, expect rearing procedures such as hand‐raising—widely used in ethology and socio‐cognitive science—to alter the development of individual social behaviour. We investigated how the rearing style later affected (a) variation in relationship strength among peers and (b) individuals’ patterns of social interactions, in three captive groups of juvenile non‐breeders consisting of either parent‐raised or hand‐raised birds, or a mix of both rearing styles. In the three groups, irrespectively of rearing style: strongest relationships (i.e., higher rates of association and affiliations) primarily emerged among siblings and familiar partners (i.e., non‐relatives encountered in early life), and mixed‐sex and male–male partners established relationships of similar strength, indicating that the rearing style does not severely affect the quality and structure of relationships in young ravens. However, compared to parent‐raised ravens, hand‐raised ravens showed higher connectedness, i.e., number of partners with whom they mainly associated and affiliated, but formed on average relationships of lower strength, indicating that social experience in early life is not without consequences on the development of ravens’ patterns of social interaction. The deprivation of parental care associated with the presence of same‐age peers during hand‐raising seemed to maximize ravens’ propensity to interact with others, indicating that besides parents, interactions with same‐age peers matter. Opportunities to interact with, and socially learn from peers, might thus be the key to the acquisition of early social competences in ravens.

## Graphical abstract

In ravens, rearing style (parent‐ vs. hand‐raised) does not severely affect the quality and structure of relationships: strongest relationships (i.e., higher rates of association and affiliations) primarily emerge among siblings and familiar, and mixed‐sex and male–male partners establish relationships of similar strength. However, hand‐raised ravens show higher connectedness compared to parent‐raised, i.e., number of partners with whom they mainly associated and affiliated, indicating that social experience in early life is not without consequences on the development of ravens’ social behaviour.

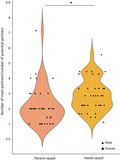

## 1 INTRODUCTION

The way individuals integrate into their social environment can have major implications for their health, survival and reproductive success (Cameron, Setsaas, & Linklater, 2009; Schülke, Bhagavatula, Vigilant, & Ostner, 2010; Silk, 2007; Stanton & Mann, 2012). Relationships can compensate part of the costs associated with group living, for instance by regulating conflicts (Preuschoft & van Schaik, 2000), facilitating access to resources (Jolles, Ostojić, & Clayton, 2013; Scheiber, Weiß, Frigerio, & Kotrschal, 2005) or the transfer of information (Allen, Weinrich, Hoppitt, & Rendell, 2013; Kawai, 1965; Schwab, Bugnyar, Schloegl, & Kotrschal, 2008). Therefore, the social environment acts as a strong selective force on behavioural traits and cognitive abilities allowing individuals to better navigate social interactions. Large brains and enhanced cognitive abilities are thought to have evolved in response to the constraints and challenges generated by the social environment (Dunbar, 1992; Humphrey, 1976; Jolly, 1966; Whiten & Byrne, 1988). Indeed, group members rely upon various social and cognitive skills to form and maintain relationships (e.g., individual recognition, memory, inhibition control or transitive inferences; Dunbar & Shultz, 2007; Wascher, Kulahci, Langley, & Shaw, 2018).

In this line, social competence has recently been defined as an individual's ability to “optimize the expression of its social behaviour as a function of the available social information” (Arnold & Taborsky, 2010; Oliveira, 2009; Taborsky & Oliveira, 2012). Social competences are expected to vary across individuals through heritability, but also in response to environmental conditions through epigenetic processes (Champagne, 2008; Oliveira, 2009; Taborsky, Arnold, Junker, & Tschopp, 2012). In particular, early‐life social experiences are thought to mediate the expression and development of social behaviour throughout an individual's life in a diverse range of species, including humans (e.g., Stormshak, Bellanti, & Bierman, 1996). This has notably been corroborated by the deleterious consequences of early‐life social deprivations—for example parental care—later resulting in increased aggressiveness (in fish: Arnold & Taborsky, 2010; in rodents: Toth, Mikics, Tulogdi, Aliczki, & Haller, 2011), lower propensity to gregariousness and poorer social integration (in apes: Kalcher‐Sommersguter et al., 2015; in fish: Hesse & Thünken, 2014), or altered emotional responsiveness (in apes: Clay & de Waal, 2013; in rodents: Branchi & Alleva, 2006).

Hence, we can expect specific rearing treatment, such as hand‐raising, to affect the development of individual's social behaviour and competences. Hand‐raising is used for a variety of purposes, from animal husbandry in agriculture, production of pets, to the conservation of endangered populations. It is also used in academical research, in particular in ethology and cognitive science as it produces tamed individuals, showing reduced neophobia and fear towards humans (Adams, Cockrem, Taylor, Candy, & Bridges, 2005; Jones & Waddington, 1992). This is a first indication that hand‐raising is certainly not without consequences on the development of individual behaviour (but see Hemetsberger, Scheiber, Weiß, Frigerio, & Kotrschal, 2010). However, its effect has not been systematically investigated, and very little is known about its potential effect on the development of individual's social behaviour.

Hand‐raising deprives individuals from parental care and expose them to humans—acting as foster parents—from an early age, potentially impairing the acquisition of social skills, behaviours and specific foraging techniques (e.g. tool use) that may be socially transmitted between parents and juveniles. However, interactions with other peers—siblings and non‐related peers—also seem to play a role in the development of social behaviour (in mammals: Kempes, Gulickx, Daalen, Louwerse, & Sterck, 2008; Branchi et al., 2013; in birds: White, Gersick, Freed‐Brown, & Snyder‐Mackler, 2010; Ruploh, Bischof, & Engelhardt, 2012; Ruploh, Bischof, & Engelhardt, 2014). Indeed, we can expect a heterogeneous social environment during early life to increase the opportunities for individuals to develop their social competences, by generating diverse sets of social situations, partners and behaviours. Accordingly, the negative effect of parental care deprivation through hand‐raising might be compensated by the presence of siblings or other peers. Actually, various factors of the early social environment (e.g., type of parental care, presence of siblings and/or other non‐related peers), certainly play a role in the development of individual social behaviour. Yet, for most species, we still lack crucial insights on the relative contributions of the different components of their social systems (i.e., social organization, mating and care system, social structure; Kappeler & van Schaik, 2002), in shaping the development of individual social behaviour.

In the last decades, a growing number of studies started to investigate avian social systems and socio‐cognitive skills, and among them, corvids have received particular attention. Large‐brain birds, typically forming long‐lasting pair bonds (Emery, Seed, Bayern, & Clayton, 2007) but at the same time display a wide range of social organizations (e.g., territorial, colonial, cooperative breeders: Coombs, 1978; Goodwin, 1976), are indeed excellent candidates to investigate the evolution of sociality and social cognition. Most corvids species show an extended developmental period before becoming nutritionally independent from their parents (Coombs, 1978; Goodwin, 1976). Moreover, they can learn from others (Fritz & Kotrschal, 1999; Kenward, Rutz, Weir, & Kacelnik, 2006), in particular their parents (Holzhaider, Hunt, & Gray, 2010), and previous experiences can later affect their behaviour and strategies when facing social or ecological challenges (Emery & Clayton, 2001). We can therefore expect their very first weeks of life to be particularly crucial for the development and acquisition of their species‐specific social skills and repertoire. So far, the vast majority of studies on corvids’ social behaviour (von Bayern, Kort, Clayton, & Emery, 2007; Boucherie, Mariette, Bret, & Dufour, 2016; Boucherie, Poulin, & Dufour, 2018; Bugnyar & Kotrschal, 2002; Dally, Clayton, & Emery, 2008; Emery et al., 2007; Fraser & Bugnyar, 2012; Gwinner, 1964; Izawa & Watanabe, 2008; Kondo, Izawa, & Watanabe, 2012; de Kort, Emery, & Clayton, 2006; Logan, Emery, & Clayton, 2013; Scheid, Schmidt, & Noë, 2008) and socio‐cognitive abilities (Bugnyar & Heinrich, 2005; Bugnyar & Kotrschal, 2004; Bugnyar, Reber, & Buckner, 2016; Emery & Clayton, 2001; Heinrich, 1995; Range, Bugnyar, Schlögl, & Kotrschal, 2006; Scheid & Noë, 2010; Schmidt, Scheid, Kotrschal, Bugnyar, & Schloegl, 2011; Seed, Clayton, & Emery, 2008) have been conducted on hand‐raised individuals. However, to our knowledge, the potential effect of hand‐raising on the development of corvids’ social behaviour has never been thoroughly investigated.

Among corvids, common ravens (*Corvus corax*) emerge as a promising species to study early environmental effects on avian social behaviour. Ravens are renowned for their advanced cognitive and social skills in captivity and in the wild (Boucherie, Loretto, Massen, & Bugnyar, 2019; Güntürkün & Bugnyar, 2016). In addition, they show relatively long lifespans, as well as extended juvenile periods and parental care. In the first months after fledging, juveniles interact almost exclusively with their siblings and parents, who form long‐term monogamous pair bond and defend large breeding territories (Marzluff & Heinrich, 1991). Juveniles gradually join non‐breeder groups during summer, where they start interacting with a more diverse set of conspecifics: same‐age peers, older non‐breeders and occasionally adult pairs (Braun & Bugnyar, 2012; Heinrich, 1989). Ravens stay and rely on those non‐breeder groups as long as they have not established in a territory, which can take several years (minimum 3, sometimes >10 years, depending on the availability of breeding territories; own unpubl. data). Non‐breeder groups facilitate the access to resources, for example when those are monopolized by breeders (Marzluff & Heinrich, 1991); but it also generates competition for resources and for status (Heinrich & Pepper, 1998), fostering cognitively sophisticated solutions such as deceptive manoeuvres for keeping/pilfering cached food (Bugnyar & Heinrich, 2005; Bugnyar & Kotrschal, 2002) and third‐party interventions (Massen, Szipl, Spreafico, & Bugnyar, 2014; Szipl, Ringler, & Bugnyar, 2018). Furthermore, non‐breeder groups have an open character with individuals joining or leaving others on a daily basis; however, some individuals may meet regularly over months and even years at the same foraging sites or night roosts (Braun & Bugnyar, 2012). This variation in fission–fusion dynamics likely results in different degrees of familiarity between individuals and the necessity to regularly update social information (Loretto et al., 2017; see also Aureli et al., 2008). Thus, considering the challenges of the non‐breeder social life, we can expect the basic building blocks of ravens’ social behaviour to develop prior to the transition to non‐breeders, that is family phase.

In this study, we aimed to investigate how the rearing conditions experienced in early life later affect the development of ravens’ social behaviour in their first year. In particular, we investigated how the absence of parental care could be compensated by the presence of same‐age non‐related peers, comparing individuals raised in a family unit by their parents, to individuals collectively hand‐raised by humans, with other peers. We made use of the fact that in the past 10 years we worked with several groups of juvenile ravens at our research facility, whereby individuals could have different rearing background. We thus picked three groups of juvenile non‐breeders, characterized by different ratios of individuals with regard to their rearing history (group A: parent‐raised, group B: parent‐ and hand‐raised, group C: hand‐raised) and investigated whether differences in rearing affected the ravens’ social behaviour. Specifically, we investigated how the rearing style later affected variations in relationship strength among peer partners and individuals’ patterns of social interactions. In more details, we first compared at the group level, i. how relationship strength was affected by partners’ identity (i.e., kin, familiar, unfamiliar) and sex in the three different groups. This question was inspired by the finding of a previous study on hand‐raised juvenile ravens (a fourth group), showing that relationships were of higher quality among kin and male–male and mixed‐sex dyads (Fraser & Bugnyar, 2010b). We were interested whether these patterns could be found again despite differences in group composition due to rearing style. We then investigated at the individual level, ii. how rearing affected individuals’ patterns of interaction (i.e. number of main partners, and individual frequencies of interactions: spatial associations, affiliative and agonistic interactions). In case parental care would be most critical for the development of ravens’ social behaviour, we would expect to find substantial differences between groups and between parent‐ and hand‐raised individuals. Alternatively, if the presence of other peers compensates for the absence of parental care, we would expect: (a) similar patterns of variations in the three groups; and (b) similar individuals’ patterns irrespective of rearing style.

## 2 METHODS

### 2.1 Subjects, groups and housing

Study subjects were 30 juvenile ravens, housed in three captive non‐breeder groups between September to July in 2010–2011 (group A), 2011–2012 (group B) and 2012–2013 (group C), at the Haidlhof Research Station, Bad Vöslau, Austria. Out of 30 ravens, 14 were parent‐raised (PR, members of group A and B), and 14 were hand‐raised (HR, groups B and C) and two females were singly hand‐raised by private owners (SHR, As and Jy, group B; as their rearing history differs from all others, they were not integrated in the second part of the analysis on individual behaviour). We selected birds to be parent‐ or hand‐raised according to the years of hatching, i.e., we wanted to establish a non‐breeder group composed of parent‐raised birds in 2010 and a non‐breeder group of hand‐raised birds in 2012; the mixed group of parent‐ and hand‐raised birds in 2011 was not planed but the result of ad‐hoc keeping and future breeding considerations.

**Figure 1:**
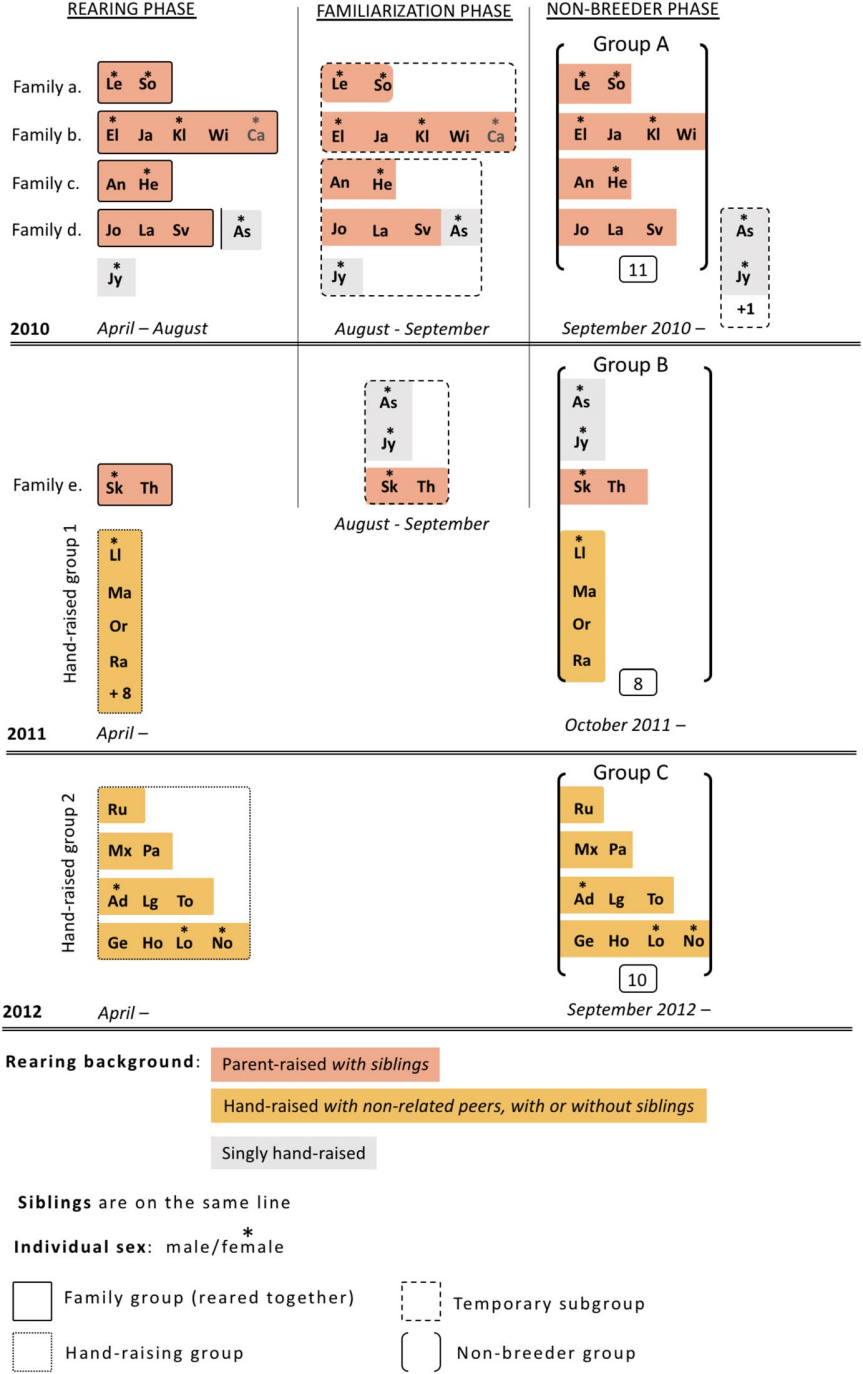
Schematic representation of groups' composition and individuals' rearing history [Colour figure can be viewed at wileyonlinelibrary.com]

All parent‐raised individuals were raised by their parents together with their siblings (with 1, 2 or 4 siblings), while hand‐raised individuals were all raised by humans with non‐related peers, and with or without their siblings (with 0, 1, 2 or 3 siblings). Individuals that were reared together (parent or hand‐raised) were kept together in the non‐breeder phase (Figure 1). All three groups were formed between mid‐September and early October of each year. They were composed of (a) group A, 12 individuals—6 males and 6 females; (b) group B, 8 individuals—4 males and 4 females; and (c) group C, 10 individuals—7 males and 3 females. All but two subjects hatched during the breeding season preceding their integration in the non‐breeder group, around April (As and Jy, group B, hatched one year earlier; Figure 1). Thus, in each group, except two individuals in Group B, all juveniles had the same age. All groups were housed in large outdoor aviaries (group A: 225 m^2^; group B: 192 m^2^; group C: 192 m^2^) containing wooded perches, trees, branches, tree trunks, platforms, stones, and shallow pools for enrichment and bathing. The ground was covered with sand, stones and woodchips for caching. Birds were fed twice a day with a mixture of different meats, vegetables, fruits, grains and yogurt, and had ad libitum access to water. All birds were identified by coloured leg rings.

### 2.2 Rearing history

#### 2.2.1 Parent‐raised individuals

PR ravens came from four captive breeding pairs in 2010 (Alpenzoo Innsbruck, Austria: 2 offspring; private owner in Klosterneuburg, Austria: 2; Zoo Wels, Austria: 3; Nationalpark Bayrischer Wald, Germany: 5) and one pair in 2011 (Konrad Lorenz Research Station Grünau, Austria: 2 offspring). PR ravens stayed with their parents and siblings in separated aviaries for 20 weeks in 2010 and 18 weeks in 2011 (rearing phase; April to August). Then, before integrating their non‐breeder group, all PR ravens were temporarily grouped with a subset of non‐related peers from their future non‐breeder group (familiarization phase; Figure 1). Doing so, both parent‐ and hand‐raised individuals encountered non‐related peers before joining their non‐breeder group (parent‐raised: 2, 3 or 5; hand‐raised: 6, 7, 8, 9, 11 non‐related peers; Figure 1). In particular, in 2010, we formed two temporary subgroups for 4 weeks by grouping in each subgroup, siblings from two different families together (August to mid‐September; Figure 1). The two subgroups were housed in adjacent aviaries (each 10 × 8 × 5 m). One of the two subgroups included also the two singly raised ravens As and Jy. These two singly raised birds did not join the main non‐breeder group of that year to prevent them from injuries due to frequent conflicts. In 2011, they served as non‐related peers for the two PR siblings from KLF Grünau for 6 weeks (August to the end of September) and were later integrated in this year's non‐breeder group. As a result, groups A and B were composed of different ratios of kin (i.e. same year siblings) and familiar individuals (i.e., non‐relatives encountered prior to the non‐breeder phase; Figure 1). Note that from the five PR siblings of Bayrischer Wald in 2010, only four could be considered in this study as one female (Ca) escaped from the aviary shortly after her introduction in the non‐breeder group.

#### 2.2.2 Hand‐raised individuals

HR ravens were taken out of the nest at around three weeks of age and raised to fledging by human foster parents (end of April – early May). In 2011, we obtained four genetically non‐related birds originated from wild nests in Southern Sweden and reared at the Corvid Cognition Station, University Lund, together with eight additional juveniles (hand‐raised group 1; Figure 1). They were thus exposed to 11 non‐related peers during rearing. Our four birds were transferred to Haidlhof Research Station, Austria, in early September and fused with the subgroup consisting of two PR and two the SHR birds in early October (non‐breeder group B). In 2012, we obtained 10 chicks from four nests of captive breeding pairs (Wildpark Haag and Wels zoo, Austria; Nationalpark Bayrischer Wald, Germany; Gymnasium Spanga Stockholm, Sweden), one containing a single individual, and the three others containing: two, three and four siblings. We kept this nestling composition during hand‐raising at Haidlhof, with siblings sharing an artificial nest box. Upon fledging, young ravens started intermingling between nests and sibling groups. Thus, from that time on, they were exposed to either 6, 7, 8 or 9 non‐related peers (Figure 1). As group C was only composed of hand‐raised individuals kept together since rearing, they were all familiar to one another.

#### 2.2.3 Singly hand‐raised individuals

The two SHR birds were originated from one wild nest (Jy) and one captive breeding pair in Wels Zoo (As), in 2010. They were singly hand‐raised to fledging by private persons, thus without any contact with their parent and siblings. Both SHR arrived at Haidlhof Research Station almost at the same time as the PR of that year. Hence, they were first grouped with five PR ravens (see above), among which were As's three genetical siblings. After 2 weeks, we took the two SHR birds out of the peer group and housed them together with an adult female for approximately one year, which strongly facilitated their way of interacting with conspecifics. We could thus put them in a subgroup with the two PR siblings from 2011 and eventually integrate them in non‐breeder group B (Figure 1).

#### 2.2.4 Animal welfare note

The ravens were obtained and housed in accordance with Austrian Law and local government guidelines. They remained in captivity after the completion of this study for further research at the University of Vienna, Austria. As the study was non‐invasive and based purely on behavioural observations, it was not classified as animal experiment in accordance with the Austrian law (§ 2. Federal Law Gazette No. 501/1989). It was approved as part of a larger study on raven social cognition by the ethical board of the behavioural research group at the Faculty of Life Sciences, University of Vienna (Nr: 2015–003a).

### 2.3 Data collection

#### 2.3.1 Studied periods and observational protocols

For all groups, data were collected between October and July. Data collection was divided into three periods according to ravens’ natural life cycle: (a) period 1 (P1), from October to December, corresponds to the integration of yearly juveniles’ in wild non‐breeder groups after they left family units at the end of the summer; (b) period 2 (P2), from January to March, corresponds to the breeding period and its onset for adult breeding pairs; and (c) period 3 (P3), from April to August, typically corresponds to the family stage from hatching to fledging of offspring, which again goes along with hormonal changes also detected in young birds (own unpubl. data). This subdivision of the data set also ensured a balance between the quantity of data and the temporal relevance of the social patterns extracted per period. Note that three individuals had to be removed from group C in course of the study, after they received severe aggressions from other group members (Mx, Pa, Ru). Group C was thus composed of 10 individuals in P1 and P2, and 7 individuals in P3. For each group and each period, we worked on a total of (a) 20 (P1), 21 (P2) and 16 (P3) observation sessions for group A; (b) 18, 22 and 25 for group B; and (c) 24, 19 and 21 for group C. During each observation session, all group members were systematically observed, in a random order, using 5‐min individual focal sampling (Altmann, 1974), resulting in a total of 1,724 focal protocols for all groups and periods. Observation sessions were performed once a day, using video recording from outside of the aviaries (i.e., Canon LEGRIA HF S20). All videos were coded using Solomon Coder (© by András Peter). To ensure inter‐coder reliability, all coders were trained on a subset of videos, previously coded by a single coder who served as a reference for all others. Coders had to reach at least 85% of concordance with the coding of reference before starting.

#### 2.3.2 Social behaviours

We defined and grouped functionally equivalent behaviours as follows: (a) spatial associations: (i) spatial proximities (i.e., two individuals located 0 to 1 m apart on the ground or at perch, for more than three seconds); and (ii) contact‐sit (i.e., two individuals sitting within one body's length, typically at perch, for more than three seconds); (b) affiliative interactions: (i) allopreening (i.e., one subject runs its beak through the feathers of another bird and/or touches any part of its body with its beak); and (ii) co‐manipulations (i.e., co‐feeding, food sharing, object co‐manipulation, offering of food/object and transfer of food/object); and (c) agonistic interactions (i.e., chase, peck, threat, displacements). See the complete ethogram in supplementary material. For each focal protocol, all behaviours were coded continuously, either as events (e.g., for agonistic interactions) or duration (when relevant e.g., for spatial association). However, we only considered behavioural frequencies and not durations in the analyses. Also, because the directionality was not available for all behaviours, we only worked on undirected data.

### 2.4 Data analyses

#### 2.4.1 Relationship strength: Sociality index

We used a dyadic undirected sociality index—based on Silk, Altmann, and Alberts (2006) and applicated by Boucherie et al. (2016) on an avian model (rooks, *Corvus frugilegus*)—to evaluate the relative strength of relationships in each group and each period. We used spatial proximities (PP), contact‐sit (CS), allopreening (AP) and co‐manipulations (CM) as follows:

$$S_{ij,x}=\left(\left(PP_{ij,x}/PP_{x}\right)+\left(CS_{ij,x}/CS_{x}\right)+\left(AP_{ij,x}/AP_{x}\right)+\left(CM_{ij,x}/CM_{x}\right)\right)/4$$

with *PP_ij,x_* the dyadic frequency of spatial proximity for the dyad *ij*, in the period *x*, divided by *PP_x_*, the mean frequency of spatial proximity for all potential dyads in the group, in period *x* (and similarly for CS, AP and CM). The denominator is fixed and refers to the number of variables. The value of the sociality index increases with the strength of the relationship.

#### 2.4.2 Identification of individual's main partners

**Figure 2:**
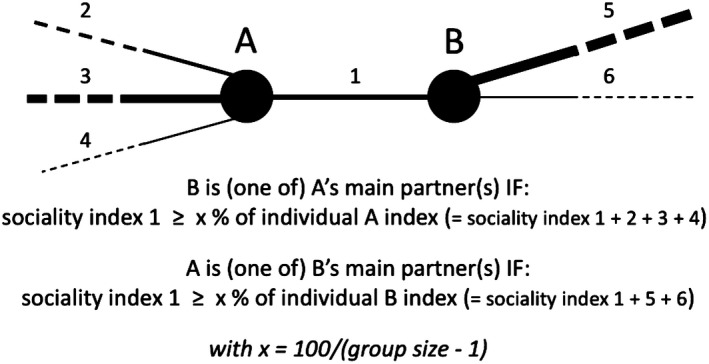
Schematic representation of the rule followed for the identification of individuals’ main partner(s), for two theoretical individuals A and B, respectively, involved in four and three relationships (numbered from 1 to 6), relationship n°1 being shared by A and B

In each group and period, we aimed to identify individuals’ main partners, by considering the relative strength that each relationship represented for both partners with respect to their own social network of relationships. To do so, we computed individual indices, summing for each individual the sociality indices for all relationships in which it was involved. Then, for an individual A, B was considered as (one of) its main partner(s) in case the sociality index of the relationship it shared with B represented more than, or equalled, *x* % of its individual index, with *x* = 100 / (group size – 1) (Figure 2). Following this, in a group composed of 11 members, an individual could have a maximum of 10 main partners, in case it is involved in relationships of equal strength with all group members (i.e., the sociality indices of all its relationship would represent 10% of its individual index).

### 2.5 Statistical analysis

#### 2.5.1 Patterns of relationship strength variations: effect of partners’ identity and sex across groups

We first aimed to compare how differences in group composition due to rearing style affected the patterns of relationship strength variation in the three groups. In more details, we investigated whether relationship strength (i.e., dyadic sociality index, response variable) varied similarly according to (a) the identity (sibling, familiar, unfamiliar) and (b) the sex of peer partners (mixed‐sex, female–female, male–male) in the three groups, thus despite differences in group composition, that is in particular here, individuals’ rearing background. To do so, for each group separately (considering all periods), we ran a generalized mixed model with a negative binomial and a log‐link function, with the dyadic sociality index as response variable (GLMM; function “glmer.nb” in R package lme4 v.1.1–13; Bates, Mächler, Bolker, & Walker, 2014). We used the identity and sex of partners as fixed factors and as random factors: the identity of the dyad and the period (used a categorical variable). Note that in group C all individuals were familiar to one another, partners’ identity was therefore reduced to two levels: sibling and familiar.

#### 2.5.2 Individual patterns of social interaction: number of main partners and frequencies of interactions

We then investigated how (a) individual rearing style (parent, hand‐raised) and (b) the number of siblings present during rearing (used as a continuous numerical variable: 0, 1, 2, 3 or 4) affected the number of main partners individuals had in their non‐breeder group (response variable). To do so, we used one generalized linear mixed model with a Poisson distribution (for all groups together). To control for group size, we fitted the number of main partners by the number of potential partners (i.e., group size minus one; using log‐transformation and an offset function). We added as random factor the period (used as a categorical variable), the identity of the individual nested in the group and individual's sex (male, female).

We then investigated how individual rearing style and the number of siblings during rearing affected individuals’ frequencies of (a) spatial association (model 1, using a LMM; function “lmer” in R package lme4 v.1.1–13; Bates et al., 2014); (b) affiliations (model 2, using a GLMM with a negative binomial distribution) and iii. agonistic interactions (model 3, using LMM). Each response variable was undirected, and obtained by summing all interactions (e.g., spatial associations) emitted and received per individual and per period. We used as an offset the number of observation sessions per periods.

Note that As and Jy (group B) were not included in this part of the analyses on individual patterns of interaction, as they were still juveniles but one year older than their other group members, and singly hand‐raised (see the Methods section on Rearing history).

#### 2.5.3 General statistics

For all models, the normality of the model’ residuals was assessed using the Shapiro–Wilk normality test (function “shapiro.test” in R package stats 3.4.0; R Core Team, 2017). To discriminate between Poisson and negative binomial regressions, we considered the dispersion of the data (negative binomial for over‐dispersed data). When used in a model, we rescaled the number of siblings (used as a continuous variable). All statistics were performed using RStudio 1.1.383 software with a significance threshold set at α = 0.05.

## 3 RESULTS

**Figure 3:**
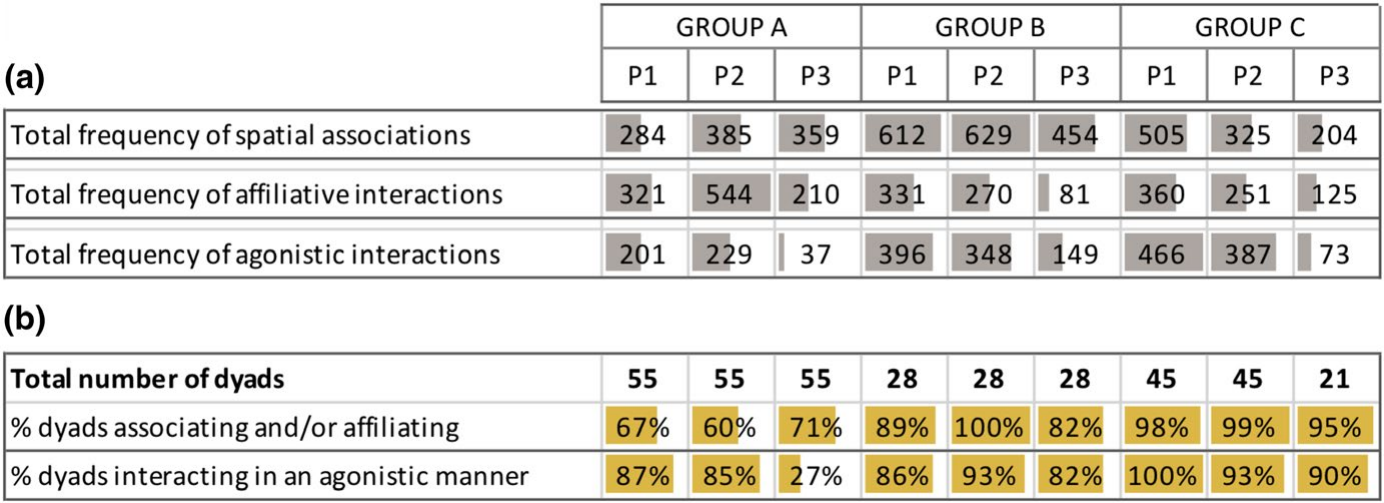
(a) Frequencies of interactions and (b) percentages of dyads interacting (at least once) across groups and periods. Data bars depict relative frequencies (grey) and percentages (gold) across groups and periods. Longer bars represent higher values. For all bars, the minimum was set to zero, and the maximum: (i) by the highest value in the row for frequency bars; and (ii) to 100% for percentage bars [Colour figure can be viewed at wileyonlinelibrary.com]

Over the three studied periods, we worked on a total of 3,757 spatial associations, 2,493 affiliations and 2,286 agonistic interactions. Across groups and periods, at minimum 60% of all potential dyads in group A, and 82% in group B and C, were recorded at least once in spatial association and/or affiliating; and at minimum 82% of all potential dyads interacted at least once in an agonistic manner (except in group A, P3; Figure 3).

### 3.1 Patterns of relationship strength variations: effect of partners’ identity and sex across groups

#### 3.1.1 Effect of partners’ identity on relationship strength across groups

Whenever groups comprised unfamiliar individuals (groups A: PR and B: mixed), they had significantly weaker relationships compared to siblings and familiar partners (Table 1). In addition, siblings had significantly stronger relationships than familiar partners in groups A (PR) and C (HR) (Table 1). They had relationships of similar strength in group B, which was, however, only composed of a single dyad of siblings (Table 1).

**Table 1:** Summary of LMM and GLMM outputs for all models investigating: (1) relationships strength, that is dyadic sociality indices; and (2) individual behaviour: (i) number of main partners; (ii) individual frequencies of spatial associations; (iii) affiliations; and iv. agonistic interactions

	relationship strength					
	Dyadic sociality indices: Partners’ sex + Partners’ identity *+ (1| Dyad) + (1|Period)*					
	Estimate	Standard error	z‐value	*p*‐value	*N*	$\bar{x}$ ± SE
GROUP A: Parent‐raised						
Intercept	2.95	0.52	5.72	<.001***		
MM versus FF	1.39	0.43	3.20	<.01**	45–30	123 ± 225–83 ± 230
Mixed versus FF	0.88	0.40	2.19	≤.05*	90–30	94 ± 220–83 ± 230
Mixed versus MM	−0.51	0.33	−1.54	.12	90–45	94 ± 220–123 ± 225
Siblings versus Familiar	2.24	0.42	5.35	<.001***	33–42	395 ± 358–60 ± 88
Unfamiliar versus Familiar	−1.56	0.34	−4.67	<.001***	90–32	11 ± 18–60 ± 88
Unfamiliar versus Siblings	−3.80	0.36	−10.46	<.001***	90–33	11 ± 18–395 ± 358
GROUP B: Mixed [Parent and Hand‐raised]						
Intercept	4.22	0.47	9.05	<.001***		
MM versus FF	1.06	0.60	1.81	.07	18–18	181 ± 236–46 ± 81
Mixed versus FF	0.37	0.50	0.74	.46	48–18	90 ± 200–46 ± 81
Mixed versus MM	−0.69	0.50	−1.40	.16	48–18	90 ± 200–181 ± 236
Siblings versus Familiar	−0.32	1.07	−0.30	.77	3–33	71 ± 56–233 ± 258
Unfamiliar versus Familiar	−2.48	0.41	−6.11	<.001***	48–33	11 ± 13–233 ± 258
Unfamiliar versus Siblings	−2.16	1.05	−2.07	≤.05*	48–3	11 ± 13–71 ± 56
GROUP C: Hand‐raised						
Intercept	1.98	0.50	3.92	<.001***		
MM versus FF	1.12	0.52	2.18	≤.05*	48–9	77 ± 165–17 ± 20
Mixed versus FF	1.65	0.51	3.24	<.01**	54–9	134 ± 173–17 ± 20
Mixed versus MM	0.53	0.28	1.91	.06	54–48	134 ± 173–77 ± 165
Siblings versus Familiar	1.96	0.31	6.35	<.001***	29–82	269 ± 243–40 ± 54

	individual behaviour					
	Number of main partners: Offset(log(number of potential partners)) + Type of rearing + scale(Number of siblings) *+ (1|Period) + (1|Group/ID) + (1|Individual Sex)*					
Intercept	−1.22	0.11	−11.45	<.001***		
Parent versus Hand‐raised	−0.32	0.16	−2.02	≤.05*	39–39	2.08 ± 1.04–2.28 ± 0.97
Number of siblings	0.06	0.08	0.80	.44		

	Individual frequency of spatial associations: Offset(log(number of observation sessions)) + Type of rearing + scale(Number of siblings) *+ (1|Period) + (1|Group/ID) + (1|Individual Sex)*					
	Estimate	Standard error	*t*‐value	*p*‐value	*N*	$\bar{x}$ * ± SE*
Intercept	112.57	34.57	3.26	<.01**		
Parent versus Hand‐raised	−51.30	26.53	−1.93	.05	39–39	70 ± 39.64–105.92 ± 59.51
Number of siblings	1.33	7.93	0.17	.87		

Individual frequency of affiliations						
	Estimate	Standard error	*z*‐value	*p*‐value	*N*	$\bar{x}$ * ± SE*
Intercept	0.82	0.28	2.92	≤.01**		
Parent versus Hand‐raised	0.05	0.26	0.21	.84	39–39	61.49 ± 42.40–57.64 ± 35.59
Number of siblings	0.21	0.13	1.60	.11		

Individual frequency of agonistic interactions						
	Estimate	Standard error	*t*‐value	*p*‐value	*N*	$\bar{x}$ ± *SE*
Intercept	55.20	21.75	2.54	≤.05*		
Parent versus Hand‐raised	−4.78	12.89	−0.37	.71	39–39	32.92 ± 30.64–71.59 ± 35.31
Number of siblings	−3.13	3.94	−0.79	.43		

Note

Note that patterns of relationships quality (1) are compared across the three groups, and therefore, the model ran separately for each group. The response variable and the lists of offset, fixed factors and random factors (in italic) are shown before the model outputs. Sample size, mean and standard error of the mean are also reported for all levels of categorical variables.

* ≤.05;

** <.01;

*** <.001.

**Figure 4:**
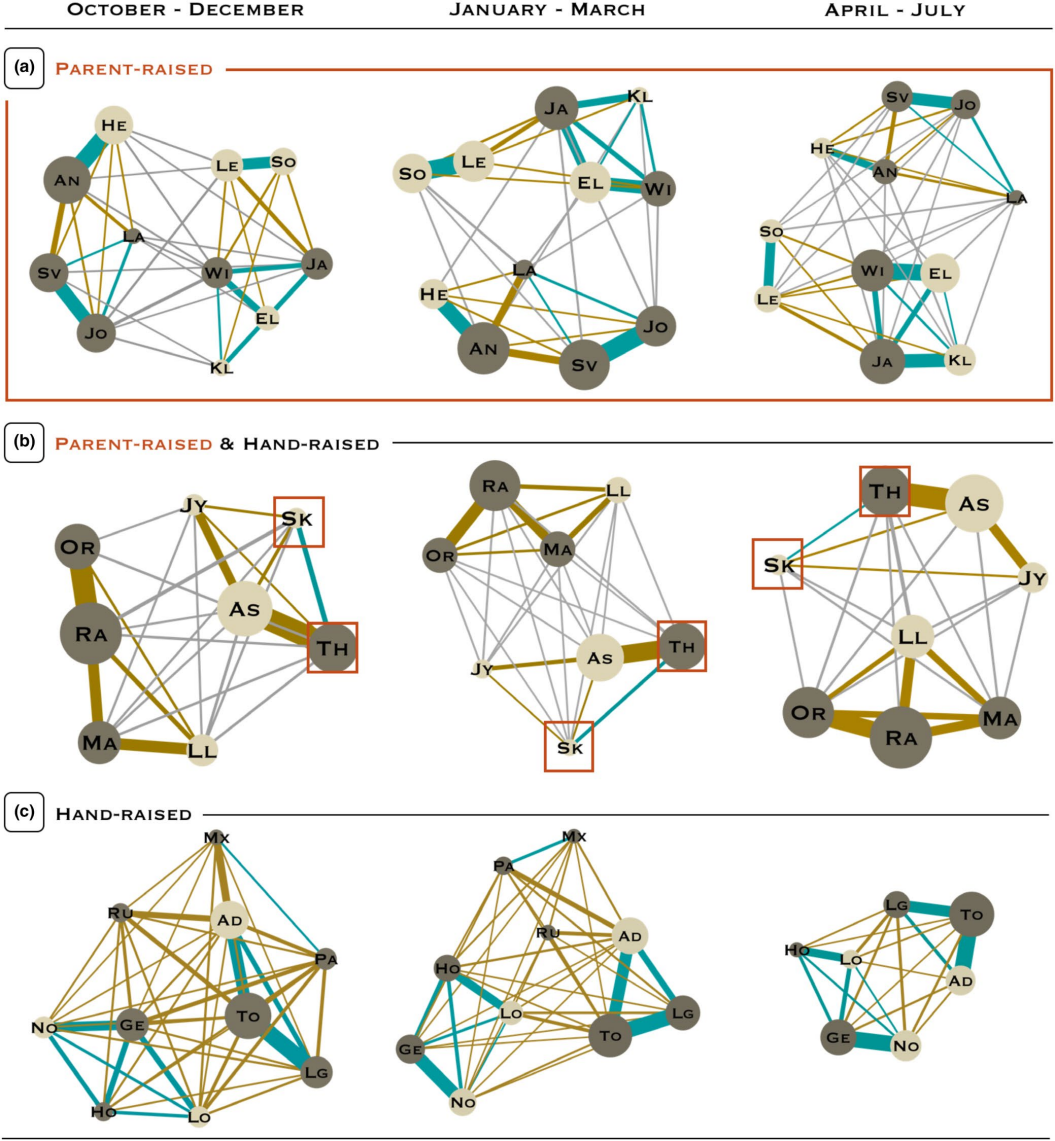
Social networks of relationships for all groups (A, B and C) and all studied periods. Nodes represent individuals and are coloured in beige for females and grey for males. The thickness of the lines indicates the strength of the relationship, based on undirected dyadic sociality indices, with thicker lines for stronger relationships. Relationships are coloured in blue among siblings, in gold among familiar individuals and in grey among unfamiliar individuals. In each network, the size of the nodes is proportional to the individual total index (i.e., sum of all sociality indices per individual), and a ratio of 3 graduate nodes from the biggest to the smallest. Parent‐raised individuals are bordered by a red square. All other individuals were hand‐raised. See Figure 1 for more details on individuals rearing background. Networks were built using the software Gephi 0.9.2 (Bastian, Heymann, & Jacomy, 2009) [Colour figure can be viewed at wileyonlinelibrary.com]

Across periods, the strongest relationship at the group level always emerged among siblings in groups A and C, but among familiar partners in group B. The unique dyad of siblings present in group B was, respectively, the 6th (P1), 7th (P2) and 13th (P3) strongest relationship of its group (over 28; Sk‐Th; Figure 4). At the individual level, the best partner (i.e. relationship with the highest social index per individual) was always either a sibling or a familiar individual. Whenever individuals had siblings in their group (irrespectively of their rearing background), they were most often the best partner, but not always: on eight occasions, a familiar individual was best partner instead of a sibling (~12% of all potential cases; Figure 4).

#### 3.1.2 Effect of partners’ sex on relationship strength across groups

In all groups, mixed‐sex and male–male partners were involved in relationships of similar strength (Table 1). In addition, mixed‐sex and male–male partners both had significantly stronger relationships than female–female partners in groups A and C (Table 1), however not in group B (Table 1). Note that the proportions of kin and familiar partners among female–female dyads were similar to those of male–male and mixed‐sex dyads: 63% of female–female dyads were either siblings or familiar females, against 76% for male–male dyads and 58% for mixed‐sex dyads.

#### 3.1.3 Individual patterns of social interaction: number of main partners and frequencies of interactions

**Figure 5:**
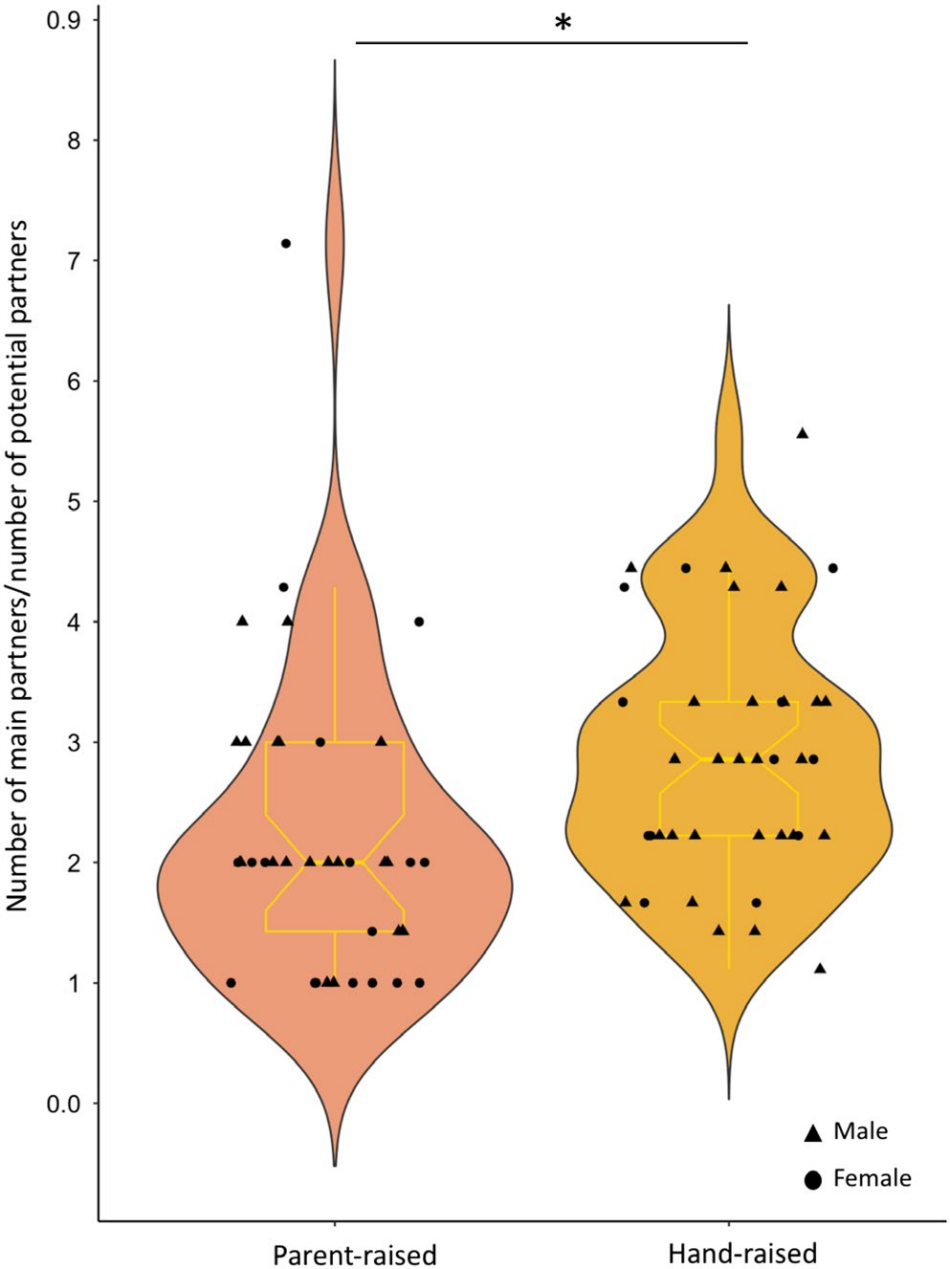
Distribution of the number of main partners (per individual and period) corrected by the number of potential partners per group and period, according to the rearing style (i.e., parent‐raised, hand‐raised). The violin plots are composed of frequency distributions (Kernel density plot) mirrored on both sides of the plot to form a symmetrical shape; and box plots. The width of each plot indicates the probability density of the data for the different y‐values. The bottom part of boxplots represents the first quartile, the top the third quartile, the thick line across the box the median, circle outliers and whiskers extend to 1.5 times the interquartile range. Sample size: 39 data points for parent‐raised individuals (11 individuals over three periods in group A, and two individuals over three periods in group B) and 39 data points for hand‐raised individuals (4 individuals over 3 periods in group B, and seven individuals over three periods and three over two periods in group C). Data points were spread horizontally to improve visibility, that is to limit overlay of individual observations, using argument “jitter.” Statistics are based on the estimates of the models. ***<.001, **<.01, *≤.05 [Colour figure can be viewed at wileyonlinelibrary.com]

Considering the total number of partners individuals could interact with, hand‐raised individuals had a significantly higher number of main partners than parent‐raised individuals (Table 1; Figure 5). The rearing style had no significant effect on the individual frequencies of affiliations and agonistic interactions (undirected i.e. emitted and received; Table 1). Descriptively, considering the estimates of the model, parent‐raised individuals appeared to be less often in spatial association with conspecifics than hand‐raised individuals. However, if the effect seems strong considering the estimates, this difference was not significant, possibly due to the limited sample size (Table 1). The number of siblings present during hand‐raising had no significant effect on the number of main partners or on the individual frequencies of spatial associations, affiliations and agonistic interactions (undirected i.e. emitted and received; Table 1).

### 3.2 Singly hand‐raised individuals

Across the three periods, As had two, one and two main partners; one with whom it was involved in the strongest relationship of its group (with Th, Figure 4). Its sociality indices ranged from: (a) period 1: 0 to 704, maximum sociality index in the group = 704; (b) period 2: 5 to 924, maximum = 924; and (c) period 2: 0 to 759, maximum = 759. Jy only had one main partner in all three periods (As, Figure 4), and its sociality indices ranged from: (a) period 1: 0 to 227, maximum = 704; (b) period 2: 5 to 137, maximum = 924; and (c) period 2: 0 to 271, maximum = 759).

## 4 DISCUSSION

Juvenile ravens were not interacting randomly, but instead adjusted their social interactions to their partners’ identity (sibling, familiar, unfamiliar) and sex. Despite differences in group composition, and in particular group members rearing background, we found similar patterns of relationship strength variation according to partners identity and sex in the three groups (group A: parent‐raised; group B: mixed; group C: hand‐raised). In particular, relationships were always stronger among kin (siblings) and familiar partners, when the group comprised unfamiliar individuals (groups A and B); moreover, in all groups, male–male and mixed‐sex partners had relationships of similar strength. In addition, in two groups over three (A and C): siblings formed stronger relationships compared to familiar partners (not in group B which, however, only comprised a single dyad of siblings); and mixed‐sex and male–male partners compare to female–female. Overall, these results corroborate the findings of Fraser and Bugnyar (2010b) concerning effects of kin and sex combination: in their group of hand‐raised ravens, kin also had relationships of higher value and female–female relationships were less secure than male–male and mixed relationships. Note that Fraser and Bugnyar’s (2010b) analysis of components of relationship quality differed from the current approach (e.g. value was a composite measure based on duration of contact‐sit and allopreening, and propensity of agonistic supports; based on Fraser, Schino, & Aureli, 2008); nevertheless, we found similar results in our groups (in particular in the parent‐ and hand‐raised groups). Hence, variation in relationship strength according to partners’ identity and sex seems to follow fairly consistent patterns in groups of juvenile ravens, and to be little affected by the rearing style.

In social groups, the formation of strong relationships among relatives can confer indirect fitness benefits to partners through kin selection (Hamilton, 1964; e.g., Silk, 2002; Hesse & Thünken, 2014). As a result, genetic relatedness often has a key structuring role on the form and dynamics taken by social structures (Hatchwell, 2009; Krause & Ruxton, 2002). Accordingly, siblings always formed significantly stronger relationships compared to unfamiliar individuals (whenever the group comprised unfamiliar individuals: A and B), and to familiar partners in the parent‐ (A) and hand‐raised (C) groups. However, kin are not always the preferential partners (e.g., Lukas, Reynolds, Boesch, & Vigilant, 2005; Wittemyer et al., 2009) and beside relatedness, familiarity often plays a major role on grouping patterns and social preferences (e.g., Griffiths, Brockmark, Höjesjö, & Johnsson, 2004; Koski, Vries, Kraats, & Sterck, 2012). Depending on the nature of past interactions, familiarity can increase partners’ propensity to reciprocate interactions (Brosnan & de Waal, 2002), acting as a feedback loop securing valuable relationships (in primates: Seyfarth & Cheney, 1984; Barrett, Henzi, Weingrill, Lycett, & Hill, 1999; Tiddi, Aureli, Polizzi Di Sorrentino, Janson, & Schino, 2011; in birds: Krams, Krama, & Igaune, 2006; Wheatcroft & Price, 2008). Aside from kinship, familiarity might facilitate the formation of close bonds with a wider diversity of partners, and in particular with non‐related partners, which in the breeding context might participate in limiting inbreeding. Accordingly, we found that relationships among familiar individuals were significantly stronger than among unfamiliar partners (present in the parent and mixed group). Also, although they had siblings in their group, ravens’ best partner was not always a sibling but a familiar individual instead. In the mixed group, familiar partners even formed as strong relationships as the (single) dyad of sibling of the group. Together, these results suggest that familiar partners can form relationships of equivalent quality compare to kin. However, because group B only comprised a single dyad of siblings, no conclusions can be made on the relative importance of familiarity and kinship in this group. More generally, the fact that in our study siblings were typically the most familiar partners of all does not allow us to disentangle kinship from familiarity. Indeed*,* beside their parents, parent‐raised ravens only interacted with their siblings during the whole rearing phase. Similarly, before hand‐raised ravens started intermingling between nests and interacting with non‐related peers, they shared a nest with their siblings. Further investigations are therefore needed to better disentangle the respective effects of kinship and familiarity in shaping social preferences in ravens (e.g. cross‐fostering experiment where non‐related nestlings are raised by foster parents).

Partners’ sex also affected relationships quality. In all groups, mixed‐sex and male–male partners had relationships of similar strength, and stronger than female–female's partnerships (significant in groups A and C, same tendency but non‐significant in group B). This confirms that juvenile ravens are able to form valuable relationships with multiple partners of both sexes, in particular males, independently of their upbringing. These results are also consistent with what is currently know about raven's social system and the function and benefits conveyed by these different types of partnerships. Ravens being long‐term monogamous, the formation of strong relationships among opposite‐sex partners evidently conveys reproductive benefits. Moreover, because non‐breeding ravens live in a social system characterized by a high degree of fission–fusion dynamics and fierce competition for resources, the formation of valuable relationships with and among males might also be advantageous. Indeed, as males usually show higher competitive abilities than females, individuals might benefit from such partnership through coalition formation and support in conflicts (Fraser & Bugnyar, 2010a, 2010b; Loretto, Fraser, & Bugnyar, 2012). Same‐sex partnerships can convey various types of benefits to partners, like facilitated access resources, space and mating, or support in conflicts (e.g. male–male alliances in birds: Duval, 2007; dolphins: Connor, Smolker, & Richards, 1992; primates: Schülke et al., 2010).

Investigating individuals’ patterns of interaction, we found that hand‐raised ravens had a significantly higher connectedness, considering the number of main partners with whom they associated and affiliated, compared to parent‐raised ravens. However, independently of their rearing history, ravens were all involved in similar frequencies of spatial associations and affiliations (emitted and received), suggesting that parent‐raised individuals had fewer main partners, but formed stronger relationships with them compared to hand‐raised individuals. First, the fact that siblings were kept together from rearing to the non‐breeder stage, and that during rearing same‐age partners were limited to siblings for parent‐raised ravens, might have later reinforced the strength and stability of their relationships. Second, when parents are present, juveniles may direct most of their attention and social interactions towards them, in particular in the first weeks of life. Hand‐raised individuals were reared collectively with numerous same‐age peers (siblings and non‐relatives). Thus, the absence of parents during hand‐raising (or all relatives for hand‐raised ravens without siblings), combined with the number and diversity of same‐age peers encountered, might have generated more diverse social‐learning opportunities. Ultimately, this may have increased hand‐raised ravens’ propensity to interact with others and to form bonds with multiple partners. Moreover, being reared collectively, hand‐raised individuals might have also faced with higher degree of competition earlier in life (e.g., for food, space, social status in the group), which was found in zebra finches (*Taeniopgygia guttata*) to increase individuals’ connectivity later in life (Brandl, Farine, Funghi, Schuett, & Griffith, 2019). Further studies should therefore examine in more details the effect of the presence versus. absence of relatives, with a closer focus on the patterns of interactions experienced in early life (e.g., diversity of social repertoire, social roles and contexts experienced). Notably, it would be very interesting to examine how parent's behaviour affect siblings’ propensity to interact with one another.

While the diversity of social partners encountered seems to be key, the time period when ravens gain their first experiences also seems to matter. Notably, if the two SHR females (Jy and As) were completely deprived of social experiences in the very first weeks, they later joined temporary groups of same‐age peers. It is then interesting to note they both formed strong relationships in their group: while Jy's only main partner was As, As for its part had a second main partner, and their relationship was the strongest of their group in all periods (As and Th). Indeed, early‐life effects are likely mediated by socio‐environmental conditions throughout (the first years of) life (Gersick, Snyder‐Mackler, & White, 2012; Ruploh et al., 2012, 2014; Sachser, Kaiser, & Hennessy, 2013; White et al., 2010). We can, in particular, expect individuals to further develop their social competence when they experience the need to use such skills. Non‐breeder aggregations are characterized by a high degree of fission–fusion dynamics, generating high diversity and unpredictability in group membership, which strongly contrasts with the size and stability of the territorial family unit. Thus, at that stage, the increased complexity of their social environment might promote the development of more advanced social strategies like third‐party intervention, support in conflicts and the use of bystander information. It is therefore important to bear in mind that in our set‐up, parent‐raised and hand‐raised individuals were exposed to different sets of partners at different time periods. Compare to parent‐raised individuals, hand‐raised individuals were indeed exposed earlier and for longer time period to other non‐related peers, which might also explain hand‐raised individuals’ higher connectivity at the non‐breeder stage. Therefore, the plasticity of the expression of individual social behaviour, as well as the dynamics of environmental influences on its development, should not be underestimated in future studies.

Finally, note that neither the rearing style nor the number of siblings affected individual agonistic interactions. This is rather surprising, as the deprivation of parent care is often found to increase agonistic behaviours in numerous species, both in frequency and intensity (Arnold & Taborsky, 2010; Toth et al., 2011; Veenema & Neumann, 2009). Yet, it provides further evidence that beside parental care, interactions with same‐age peers matter in this species, and might counterbalance the negative effect of parental care deprivation early in life. In addition, we found no significant effect of the number of siblings neither on the number of main partners nor on individuals’ frequencies of interactions. Unfortunately, the current data set did not allow further investigations on the interactions between rearing style and number of siblings or to disentangle the effect of number of siblings during rearing and their mere presence in the non‐breeder stage. Future studies should therefore aim to investigate the effect of the social experience with same‐age sibling(s) during rearing, controlling for the number of siblings present later in life.

This study shows that the rearing style (parent‐ vs. hand‐raised) does not severely affect the quality and structure of relationships according to partners identity and sex in young ravens. Nonetheless, our results suggest that the social experience made by ravens in early life is not without consequences on the development of their patterns of social interaction. In particular, we found that far from becoming more aggressive or less social, hand‐raised ravens were on the contrary more inclined to interact in a positive manner with a higher number of conspecifics than parent‐raised ravens. This indicates that aside parental care, early familiarization with same‐age peers can have strong and early effects. The multiplicity and diversity of social‐learning opportunities, rather than the identity of partners present during rearing, may thus be the key to the acquisition of social competences in ravens.

## CONFLICT OF INTEREST

The authors declare that they have no conflict of interest.
