# Supplementary material for: Effect of rearing style on the development of social behaviour in young ravens (Corvus corax)
Source: Ethology. 2020 Mar 18;126(6):595–609. doi: 10.1111/eth.13010 (PMC7317586; doi:10.1111/eth.13010)
Supplement: Supplementary file 1 [file ETH-126-595-s001.docx]

**ETHOGRAM**

Spatial associations

- **Spatial proximity:** two birds 0 - 100 cm apart on the ground or at perch, for a minimum duration of three seconds
- **Contact-sit**: two birds typically sitting at perch within one body length i.e., approximately 0 – 20 cm apart (close enough to touch the other’s body), for a minimum duration of three seconds

Affiliative interactions

- **Allo-Preening**: One bird touches / runs its beak through the feathers of another bird and/or touches any part of another bird’s body with its beak / foot.

Co-manipulations

- **Co-feeding**: two birds eat next to each other on different food items (i.e., not the same food piece) within a reaching distance (approximately 30 cm).
- **Food sharing**: two birds eat from the same food item.
- **Object co**-**manipulation**: two birds manipulate the same fixed / portable object(s), or different object(s), within a reaching distance.
- **Offering**: one bird visually presents another bird an item (food or object), within a reaching distance.
- **Transfer**: one bird in possession of an item (food or object) allows the item being taken by another bird (passive transfer) or actively gives the item to another bird (active transfer), with or without begging from the receiver before and during the transfer.

Agonistic interactions

- **Chase**: One bird pursues / rushes toward another one flying / running.
- **Peck**: One bird pecks another one with its beak. Implies physical contact.
- **Threat**: One bird threats another one, with display “thick head” display and / or visual threat (the aggressor pecks in the direction of the victim without touching it), with or without vocalizations.
- **Displacement**: One bird approaches another bird within 50 cm, the other bird retreats / moves away 1 meter or more than 1 meter, with or without vocalization, with or without receiving a threat or direct physical contact between the two birds.
